# Conversion therapy with TACE, TKIs, and ICIs for unresectable BCLC stage B and C hepatocellular carcinoma

**DOI:** 10.3389/fimmu.2025.1451965

**Published:** 2025-06-10

**Authors:** Zhuoran Wang, Cunzhen Zhang, Jianhua Yin, Nan Li

**Affiliations:** ^1^ Department of Hepatic Surgery I (Ward I) Shanghai Eastern Hepatobiliary Surgery Hospital, Navy Medical University, Shanghai, China; ^2^ Department of Epidemiology, Faculty of Navy Medicine, Navy Medical University, Shanghai, China; ^3^ Key Laboratory of Biological Defense, Ministry of Education, Shanghai, China; ^4^ Laboratory of Medical Bioprotection, Navy Medical University, Shanghai, China; ^5^ The Second Affiliated Hospital, Wenzhou Medical University, Wenzhou, Zhejiang, China

**Keywords:** hepatocellular carcinoma, TACE, anti-angiogenic therapy, anti-PD-1 antibody, conversion therapy

## Abstract

**Background and aims:**

Most Hepatocellular carcinoma (HCC) diagnoses occur at advanced stages precluding radical surgical resections. Conversion therapy offers a viable chance for patients with unresectable HCC (uHCC) to become eligible for curative surgery. Despite the application of various treatment modalities for conversion therapy, uncertainties persist regarding its efficacy. Consequently, we collected clinical data to evaluate the prognosis of TACE+TKI+ICI conversion therapy and compared it with the prognoses of other conversion therapies in the literature. We aimed to elucidate the potential superiority of triplet therapy as the optimal option among the existing conversion therapy regimens, by using this comprehensive analysis.

**Methods:**

From January, 2019, to November, 2022, we collected data from 69 patients with HCC undergoing conversion therapy with the TACE+TKI+ICI triplet therapy. Ultimately, we analyzed data from 57 patients at BCLC Stages B and C in our study. We also conducted a comprehensive literature review on conversion therapy for uHCC by searching PubMed and Web of Science databases and gathered data from 9 studies comprising a total of 560 patients.

**Results:**

The conversion and disease control rate (DCR) in our cohort reached 14.0% (95% CI, 9.4–18.6%) and 66.7% (95% CI, 60.5–72.9%), respectively. When compared to the conversion rates in the literature, the triplet therapy demonstrated significant benefits, underscoring the potential efficacy of the TACE+TKI+ICIs triplet therapy.

**Conclusion:**

Our results presented improved conversion rates in patients with uHCC following TACE+TKI+ICI triplet therapy. However, overall survival (OS) and recurrence-free survival (RFS) were similar to those of other treatment modalities in the literature.

**Clinical Trial Registration:**

ClinicalTrials.gov, identifier ChiCTR2400084896.

## 1 Introduction

Liver cancer ranks sixth in incidence among the global cancers and third in terms of mortality rates. Chinese patients represent 45.3% of newly diagnosed cases globally and 47.1% of the global liver cancer-related deaths (1). HCC, as the most common sort of liver cancer, the early stages of symptoms often are not typical due to the insidious onset and undefined epidemiological history of the disease that includes cases of HBV, HCV, or alcoholic liver cirrhosis (2, 3). Thus, 70% to 80% of patients with HCC are diagnosed at an advanced stage with surgical contraindications. The 5-year survival rate of these patients is less than 20% (4). Although radical surgical resection remains the primary treatment modality for HCC, only 20% to 30% of patients are eligible for resection (5–8). Therefore, conversion therapy provides an opportunity for curative resections in most cases. Studies have suggested that HCC, following conversion treatments such as transarterial chemoembolization (TACE), radiofrequency ablation, or radiotherapy reaches 5-year survival rates of 50–60% (9–11). Alternative conversion treatment approaches include portal vein embolization (PVE) and the associating liver partition and portal vein ligation for staged hepatectomy (ALPPS) (12). These treatments enhance the eligibility of patients for radical resection.

The relatively low rates of initial curative resections for HCC have promoted research on systemic therapies, a field that has seen substantial progress. Significant advancements have been achieved in the use of anti-angiogenic inhibitors and immunotherapy. Sorafenib and lenvatinib are considered first-line treatments for patients with advanced HCC; however, their efficacy rates have not been optimal (13, 14). Encouragingly, the combination of targeted therapy and immunotherapy for advanced uHCC, such as the T+A regimen, has demonstrated a 67.2% 1-year survival rate and a median survival time of 11.2 months (15, 16). By contrast, the overall response rate (ORR) for the regimen of tyrosine kinase inhibitors (TKIs) combined with immune checkpoint inhibitors (ICIs) has reached 36% (17). Thus, the potential for improving outcomes with combination therapies is clear (18). Methods such as systemic therapy, local treatment, increasing future liver remnant (FLR)/standard liver volume (SLV), improving liver function, and antiviral therapy have all been studied and recommended for conversion therapy in the ‘Chinese Expert Consensus on Hepatocellular Carcinoma conversion Therapy (2021 Edition)’. However, in the clinical practice, some patients display suboptimal efficacy following systemic therapy. Therefore, we implemented a kind of treatment, adhering to the Chinese Primary Liver Cancer Diagnosis and Treatment Standard, using local TACE combined with systemic therapy for conversion therapy in patients with uHCC, followed by subsequent curative surgical resection.

## 2 Methods

**Figure 1:**
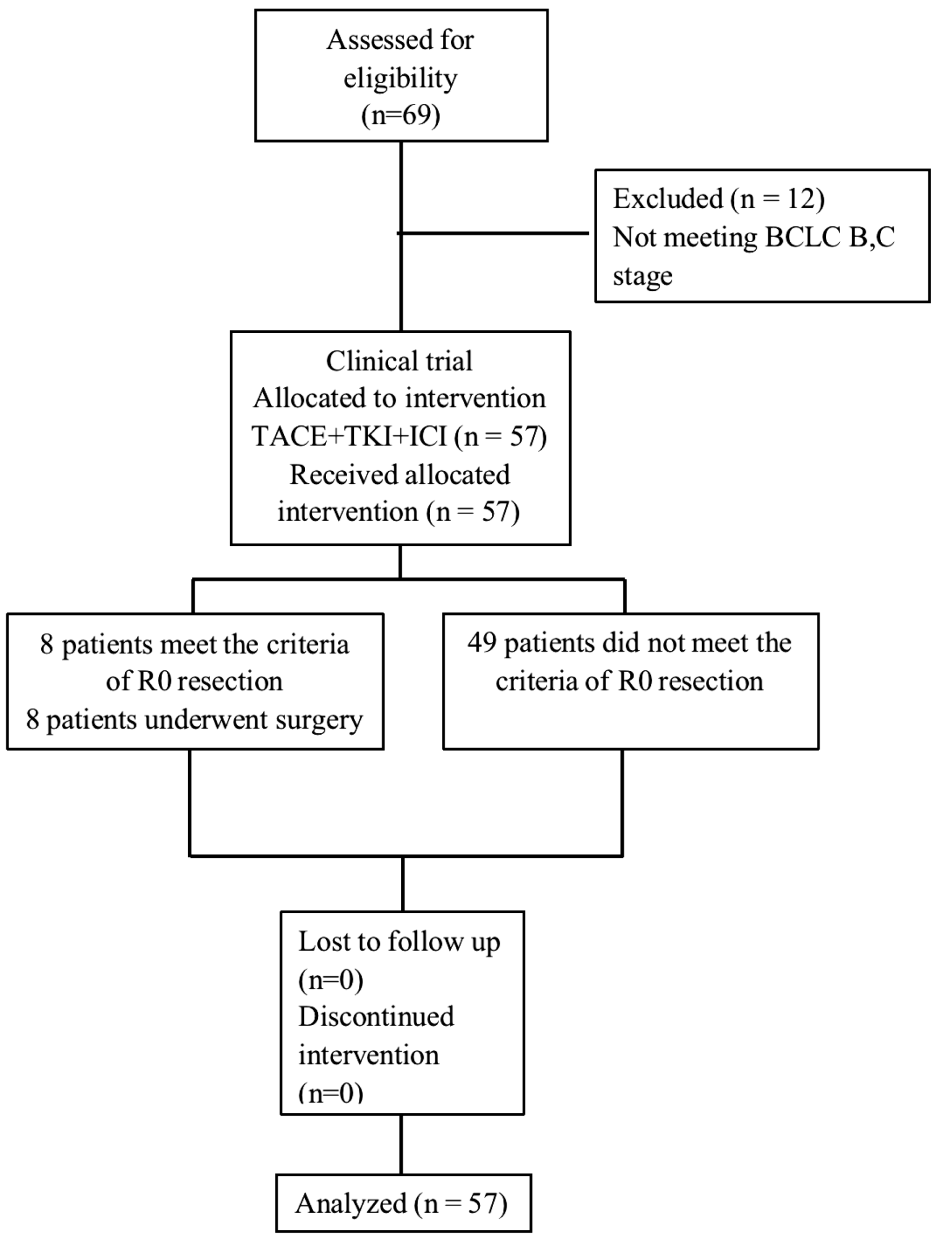
Patient flowchart.

From 2019–01 to 2022-11, we enrolled 57 patients with BCLC stage B and C uHCC at the Shanghai Eastern Hepatobiliary Surgery Hospital. Following examinations and exclusion of contraindications, these patients received TACE for local lesion control, combined with first-line targeted therapy and immunotherapy. Their HCC diagnoses relied primarily on imaging evidence of well-defined focal lesions, with evaluation based on BCLC staging and CNLC guidelines to confirm the unresectable nature of the tumors, either with or without elevated tumor markers (AFP, AFP-L3, and PIVKA). We included individuals of either sex, and those with a history of hepatitis B and ECOG performance status of 0–1. In addition, we reviewed relevant literature following a comprehensive search on Pubmed, Web of Science and Embase databases including publications up until July 2022. We found 9 articles with precise matching analyses for conversion therapy. The therapeutic interventions included TACE, targeted therapy, and immune checkpoint inhibitors (ICIs) used both solely or jointly as conversion therapies for uHCC. **Figure 1** shows the trial flow diagram and **Supplementary Figure S1** shows the flowchart for retrieving the comparison of clinical trials. The Shanghai Eastern Hepatobiliary Surgery Hospital Ethics Committee reviewed and approved the study protocol (Ethics Approval Number: EHBHKY2023-K033-P001), and we obtained signed written informed consent forms from all enrolled patients. All authors had access to the study data and reviewed and approved the final manuscript.

### 2.1 Treatment plan

Following the assessment of overall health status and liver function (Child Pugh A or B) and the exclusion of contraindications to conversion therapy, all enrolled patients initially underwent TACE. Subsequently, in the absence of significant adverse effects, the patients received oral sorafenib at a dose of 0.25 g per day, or lenvatinib (8 mg–12 mg per day, with dosing based on body weight (<60 kg: 8 mg; ≥60 kg: 12 mg)) (13, 19, 20). The targeted oral medication was administered on the same day as the immunotherapy (every 3 weeks), using drugs such as camrelizumab (21) (200 mg, every 2 weeks) or sintilimab (200 mg, every 3 weeks). We conducted regular follow-up examinations for all enrolled patients, including complete blood counts, liver, kidney, thyroid, and cardiac function tests, tumor markers assessments, electrocardiogram, and a chest X-ray before each immunotherapy session. We assessed tumor changes via periodic imaging studies, with Computerized tomography (CT) or magnetic resonance imaging (MRI) scans conducted every 6 weeks, following the RECIST v1.1 criteria for tumor response evaluation and modified RECIST standards (22, 23).

### 2.2 Surgical procedure and standards

The preoperative assessment criteria were based on blood tests and imaging data to determine whether each patient met the R0 resection standards. According to the latest international guidelines, R0 resection is defined as a microscopically margin-negative resection, with no residual tumor cells detected at the resection margin (24). Preoperative contrast-enhanced CT was utilized to further evaluate the therapeutic response and confirm eligibility for R0 resection. Conditions not meeting R0 resection standards included a residual liver volume lower than 30% in non-cirrhotic individuals, lower than 40% in cirrhotic patients, a BCLC stage class B or C, and tumor recurrence within 12 months after the initial resection. The eligibility criteria for HCC surgical resection included the feasibility of an R0 resection leaving sufficient residual volume of functional liver. Additionally, intrahepatic lesions had to exhibit at least a partial response (PR) or stable disease (SD) for a minimum of 2 months, with no severe or persistent adverse effects from systemic therapy and no contraindications for hepatic resection.

The diagnosis and grading of postoperative liver dysfunction were based on criteria proposed by the International Study Group of Liver Surgery, including an increase in the international normalized ratio and postoperative hyperbilirubinemia occurring on or after the 5^th^ postoperative day (25). We classified postoperative complications using the Clavien-Dindo system, which categorizes them based on their severity (26).

### 2.3 Tumor response and adverse reaction assessments

We assessed the tumor response using the modified Response Evaluation Criteria in Solid Tumors (mRECIST) at regular intervals (typically every 4 to 8 weeks) using contrast-enhanced CT or MRI scans to categorize responses as complete response (CR), partial response (PR), stable disease (SD), or progressive disease (PD). A third-party review committee independently evaluated the assessment results. Objective response rate (ORR) was defined as the proportion of patients achieving CR or PR, with this assessment conducted at least 4 weeks after the initial fulfillment of response criteria. The disease control rate (DCR), encompassing CR, PR, and SD, served as a broader measure of the treatment’s overall control over the disease. We extracted adverse reaction information from the hospital electronic medical records database and evaluated the data based on the Common Terminology Criteria for Adverse Events version 5.0 (CTCAE 5.0).

### 2.4 Postoperative management

After undergoing radical resection, patients returned for follow-up approximately 4–5 weeks postoperatively. We requested new preventive interventional therapies based on the pathological findings. Immunotherapy was administered continuously during this period, and targeted therapy was resumed 4–6 weeks after surgery. We re-examined the tumor marker levels before initiating the immunotherapy, with CT or MRI scans conducted every 9 weeks.

### 2.5 Data analysis

We analyzed all data using SPSS 18.0 software. OS was defined as the time from definitive diagnosis to death, and RFS was measured from the date of surgery to either the date of patient relapse or the time of patient death due to reasons other than an apparent relapse. We conducted a differential analysis of data using paired *t*-tests and exact Fisher tests. We generated Kaplan–Meier analysis OS curves, with log-rank test evaluating differences. Additionally, univariate and multivariate Cox regression analyses were performed to evaluate prognostic factors.

## 3 Results

### 3.1 Baseline characteristics

**Figure 2:**
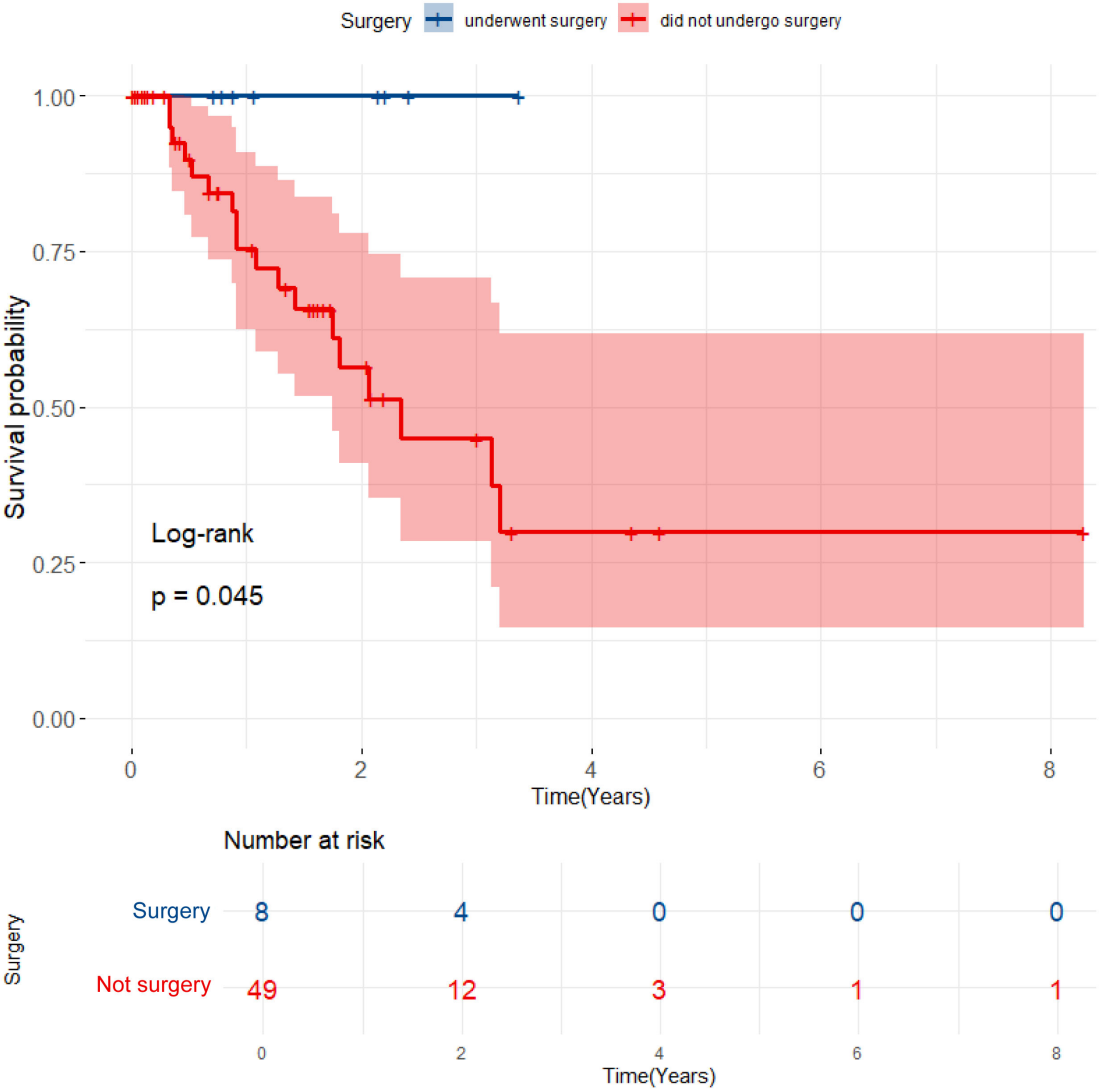
Kaplan-Meier curves for OS comparing patients who underwent surgical resection with those who did not.

As of June 5, 2024, 69 patients underwent TACE+TKI+ICIs conversion therapy for initially diagnosed uHCC at the Shanghai Eastern Hepatobiliary Surgery Hospital. Among these cases, 57 were classified as BCLC B or C, and ultimately, 8 patients underwent radical resections for primary HCC. **Table 1** summarizes the baseline characteristics of the 57 patients receiving systematic conversion therapy. Univariate Cox regression analyses on baseline variables yielded significances between AFP (*P*=0.029), albumin (*P*=0.021), and OS. After our multivariate analysis, we found significances between AFP (*P*=0.019), albumin (*P*=0.003) and OS, and between gender (*P*=0.043), abnormal prothrombin (PIVKA-II) (*P*=0.008), and RFS (**Table 2**). Patients who underwent surgical resections experienced a significant improvement in OS compared to those who did not (**Figure 2**), but we detected no significant differences in RFS (*P*>0.05). Additionally, we reviewed data from a subset of studies on HCC conversion therapy from PubMed, Web of Science and Embase. We performed a matching analysis based on BCLC staging and existing clinical data (**Table 3**). The analysis included data from various studies involving TACE, TKI, and ICIs, either single or joint. In total, 560 patients with BCLC B or C received conversion therapy, including 30 treated with TACE alone, 86 with TKI alone, 60 with a TKI+ICIs combination, 194 with TKI+TACE, and 190 with TKI+TACE+ICIs triplet therapy.

**Table 1:** Baseline characteristics.

Characteristic	Patients who underwent surgery (*n* = 8)	Patients who did not undergo surgery (*n* = 49)	*p*
Gender			0.05*
male	5	45	
female	3	4	
Age (years)			0.017*
≥53	3	40	
<53	5	9	
Etiology of HCC			1*
HBV	6	33	
non-viral	2	16	
BCLC stage			0.709*
B	4	20	
C	4	29	
Macrovascular invasion			0.002*
yes	3	0	
no	5	49	
Portal vein tumor thrombus			0.466*
yes	2	20	
no	6	29	
Hepatic vein tumor thrombus			0.644*
yes	2	9	
no	6	40	
Extrahepatic disease			0.697*
yes	2	19	
no	6	30	
Baseline AFP			1*
≥400 ng/mL	3	22	
<400 ng/mL	5	27	
Baseline PIVKA-II			0.144*
≥ 1,000 mAU/mL	6	22	
<1000 mAU/mL	2	27	
TKIs			0.464*
lenvatinib	7	46	
sorafenib	1	3	
ICIs			0.706*
camrelizumab	2	17	
sintilimab	6	32	
Baseline Total Bilirubin			0.584*
≥ 20.5 µmol/L	0	8	
<20.5 µmol/L	8	41	
ALT/AST			0.466*
<1	6	29	
≥1	2	20	
Albumin			0.580*
<35 g/L	0	6	
≥35 g/L	8	43	

*Fisher Test.

**Table 2:** Baseline characteristic univariate and multivariate Cox regression analysis with OS and RFS.

Characteristic	Overall Survival				Recurrence Free Survival			
	Univariate analysis		Multivariate analysis		Univariate analysis		Multivariate analysis	
	HR (95% CI)	*p*	HR (95% CI)	*p*	HR (95% CI)	*p*	HR (95% CI)	*p*
Gender (male/female), *n*	0.5 (0.12-2.21)	0.364	1.04 (0.18-6.16)	0.966	1.43 (0.54-3.78)	0.472	5.32 (1.05-26.88)	**0.043**
Age, years (≥53/<53)	/	/	/	/	0.7 (0.24-2.03)	0.515	/	/
Etiology of HCC (HBV/non-viral), *n*	0.85 (0.28-2.63)	0.781	1.69 (0.3-9.49)	0.550	1.24 (0.42-3.61)	0.698	2.6 (0.66-10.13)	0.170
BCLC stage (B/C), *n*	4.01 (0.92-17.46)	0.064	/	/	1.7 (0.74-3.88)	0.209	/	/
Macrovascular invasion (yes/no), *n*	/	/	/	/	0.72 (0.17-3.08)	0.653	1.82 (0.12-28.5)	0.670
Portal vein tumor thrombus (yes/no), *n*	4.92 (1.12-21.51)	0.035*	/	/	1.22 (0.56-2.65)	0.616	/	/
Hepatic vein tumor thrombus (yes/no), *n*	/	/	/	/	0.53 (0.12-2.24)	0.387	/	/
Extrahepatic disease (yes/no), *n*	1.17 (0.44-3.12)	0.757	/	/	1.4 (0.62-3.13)	0.416	2.92 (0.27-31.23)	0.376
Baseline AFP (≥400/<400 ng/mL)	0.32 (0.11-0.89)	**0.029**	0.11 (0.02-0.7)	**0.019**	0.67 (0.31-1.44)	0.308	1.32 (0.45-3.88)	0.610
Baseline PIVKA-II (≥ 1,000/<1000 mAU/mL)	1.07 (0.42-2.75)	0.882	0.52 (0.15-1.83)	0.306	0.52 (0.24-1.11)	0.093	0.24 (0.08-0.69)	**0.008**
TKIs (lenvatinib/sorafenib)	1.3 (0.29-5.72)	0.731	1.46 (0.14-15.55)	0.755	0.98 (0.23-4.21)	0.980	1.56 (0.23-10.66)	0.647
ICIs (camrelizumab/sintilimab)	1.39 (0.5-3.85)	0.522	/	/	1.1 (0.49-2.46)	0.813	0.51 (0.04-5.94)	0.591
Baseline Total Bilirubin (≥ 20.5/<20.5 µmol/L)	0.82 (0.18-3.66)	0.796	2.17 (0.3-15.63)	0.440	0.51 (0.19-1.35)	0.175	1.71 (0.47-6.19)	0.417
ALT/AST (<1/≥1)	0.7 (0.27-1.81)	0.458	0.9 (0.17-4.68)	0.896	0.51 (0.23-1.13)	0.095	0.45 (0.15-1.34)	0.152
Albumin (<35/≥35 g/L)	0.26 (0.08-0.81)	**0.021**	0.06 (0.01-0.38)	**0.003**	0.48 (0.16-1.43)	0.186	0.55 (0.16-1.84)	0.331

“/”: HR=1, no reporting of HR, confidence interval, or p-value.

**Table 3:** Matching analysis of BCLC staging between clinical studies and literature studies.

Conversion Therapy	BCLC stage	B	C	*P*
TKI+ICI+TACE	Clinical Study	24	33	
TACE	Wu (27)	17	13	0.196
TKI	He (28)	24	62	0.078
TKI+ICI	Xie (29)	17	43	0.119
TKI+TACE	He (30)	47	78	0.563
	Zheng (31)	14	15	0.157
	Cai (32)	21	19	0.312
TKI+ICI+TACE	Liu (33)	12	10	0.320
	Cao (34)	13	39	0.060
	Zheng (31)	11	11	0.527
	Cai (32)	24	17	0.108
	Teng (35)	23	30	0.891

### 3.2 TACE+TKI+ICI: conversion therapy for uHCC

In our cohort of 57 patients with uHCC, according to the mRECIST criteria, the TKI+TACE+ICIs combination therapy achieved a conversion success rate of 14.0% (95% CI, 9.4–18.6%), with an ORR of 21.1% (95% CI, 15.7–26.5%), and a DCR of 66.7% (95% CI, 60.5–72.9%). Moreover, based on mRECIST version 1.1, the independent imaging data of surgical patients revealed significant differences before and after the conversion therapy, as evidenced by the contrast-enhanced CT scans of lesions before and after treatment (**Supplementary Figure S2**). The pathological results from patients undergoing primary surgical resections consistently demonstrated favorable conversion outcomes. **Table 4** shows the baseline data of surgical patients.

**Table 4:** The data of patients undergoing surgery.

Patient number	Intrahepatic tumor size, cm	Number of intrahepatic tumors	BCLC stage	CHILD Pugh stage	Vascular invasion	TKI used	Anti-PD-1 antibody used	Tumor response, by RECIST v1.1	Tumor response, by mRECIST
1	7*6	1	C	B	M0	lenvatinib	camrelizumab	PR	CR
2	8*9	1	C	B	M0	lenvatinib	camrelizumab	PR	PR
3	12*10	1	C	B	M1	lenvatinib	sintilimab	SD	SD
4	10*9	1	B	B	M0	lenvatinib	sintilimab	CR	CR
5	14*10	1	C	B	M1	lenvatinib	sintilimab	PR	CR
6	10*8	1	B	B	M0	lenvatinib	sintilimab	PR	PR
7	12*12	1	C	B	M1	lenvatinib	sintilimab	SD	PR
8	9*8	1	B	B	M0	lenvatinib	sintilimab	PR	PR

### 3.3 TACE+TKI+ICI: a superior conversion therapy strategy for uHCC

**Figure 3:**
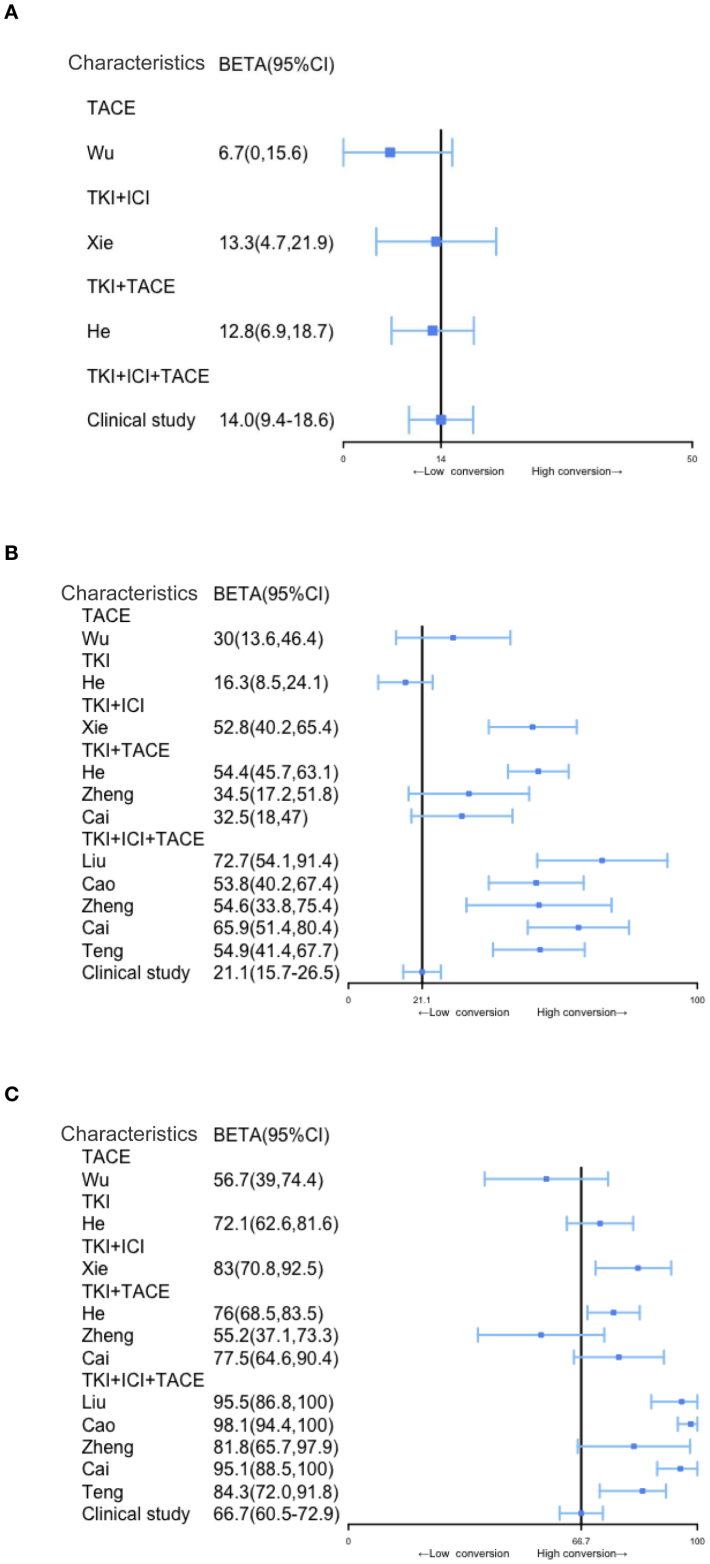
Comparison of conversion rates, ORRs, and DCRs in patients with uHCC undergoing different treatment modalities, including triple combination therapy. **(A–C)** Comparison of conversion rates, ORR and DCR among different conversion therapy modalities, the solid black line represents the clinical study.

Conversion therapies include TACE, TKI, TKI+ICI, TKI+TACE, and TKI+TACE+ICI, the conversion rate for the TKI+TACE+ICI therapy showed a distinct advantage after analyzing the conversion rates for other modalities (**Figure 3A**). However, in contrast to other studies, our clinical study finds that there are indistinct improvements in ORRs or DCRs (**Figures 3B, C**). A comparative analysis of our trial with other studies demonstrated the superiority of the TACE+TKI+ICI triplet therapy in terms of conversion rates for uHCC. However, the ORRs or DCRs of TKI+TACE+ICI in our trial not improve apparently compared with other researches.

### 3.4 TACE+TKI+ICI therapy exhibits a significant advantage in RFS for uHCC conversion therapy

**Figure 4:**
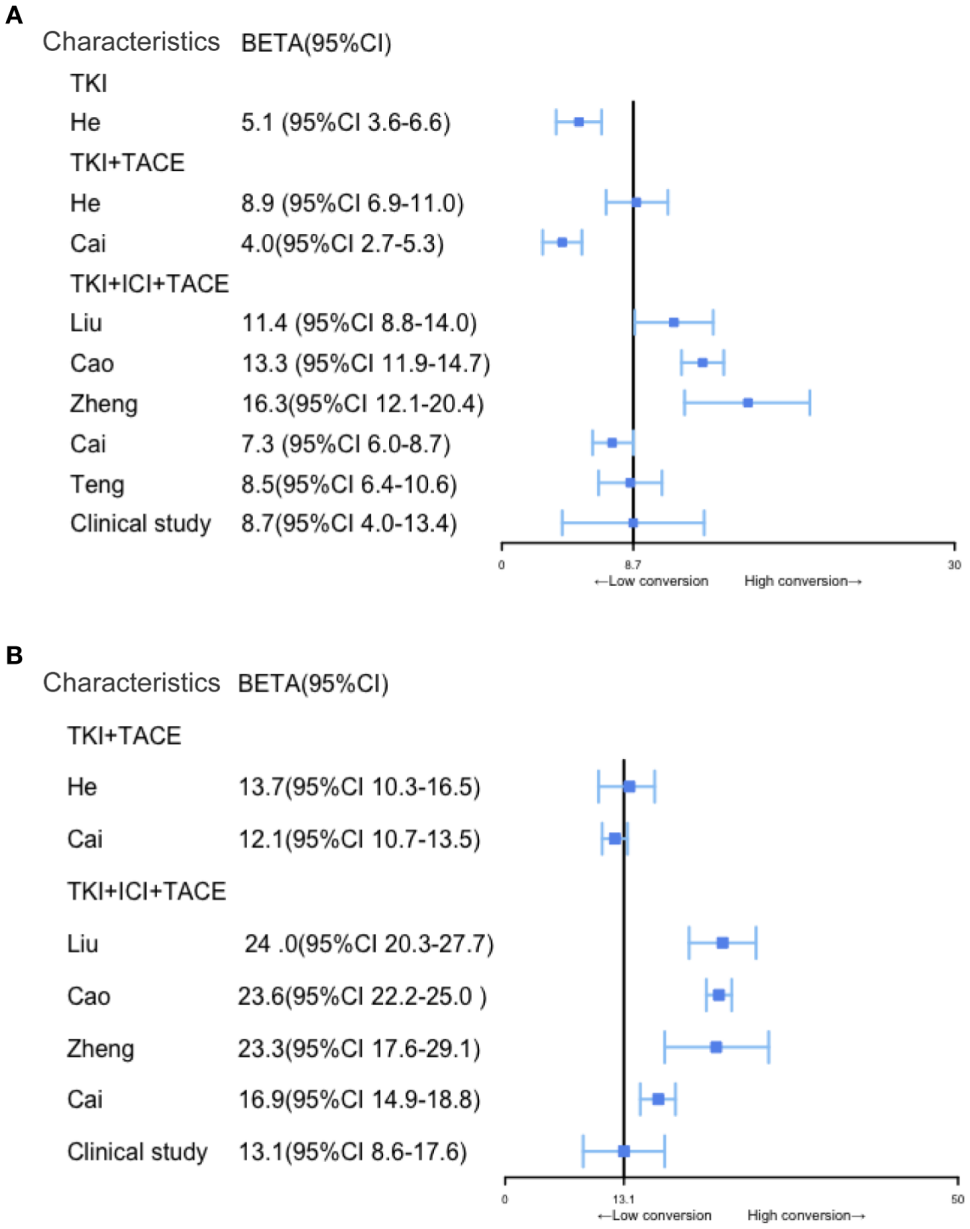
Comparison of RFS and OS among patients with BCLC stage B and C uHCC after different conversion therapy modalities. **(A, B)** Comparison of RFS and OS among different conversion therapy modalities, the solid black line represents the clinical study.

According to our clinical trial, the follow-up periods ranged from 4 to 63.3 months, with a median follow-up time of 12.1 months. During the follow-ups, 24 patients (48.9%) experienced recurrences, and 18 patients (36.7%) succumbed to the disease. The median OS was 13.1 months (95% CI, 8.6–17.6), and the median RFS was 8.7 months (95% CI, 4.0–13.4). Interestingly, in our clinical study, RFS and OS demonstrated less pronounced improvements after the conversion therapy than those reported in the literature. However, the OSs and RFSs of studies on various conversion therapies have consistently shown that triplet therapy provides significantly longer survivals than other conversion treatment modalities (**Figure 4**).

### 3.5 The adverse events with TACE+TKI+ICI therapy are manageable

In this clinical trial, we analyzed data from 57 adverse events (AEs) (100%) that occurred after triple therapy within 1 year. Grade 3 AEs occurred in 4 cases, including elevated AST in 2 patients (3.5%), leukopenia in 1 (1.8%), and severe anemia in 1 (1.8%). No adverse events exceeding grade 3 were observed, and the others were grade 2 or grade 1 AEs. After 1 year, 17 patients died, and the remaining patients had grade 2 or grade 1 AEs. We found no instances of grade 3 or above complications. Common AEs included decreased appetite, abdominal pain, elevated AST and/or elevated ALT levels, and leukopenia (**Table 5**). All AEs were assessed as mild and manageable during the follow-up periods.

**Table 5:** Adverse events of TACE+TKI+ICI treatment (CTCAE 5.0).

Adverse Event	Any Grade		Grade1-2		Grade3	
	≤ 1 year (*n* = 57)	> 1 year (*n* = 30)	≤ 1 year (*n* = 57)	> 1 year (*n* = 30)	≤ 1 year (*n* = 4)	> 1 year (*n* = 0)
Decreased appetite	28 (49.0%)	15 (50.0%)	28 (49.0%)	15 (50.0%)	0	0
Hepatalgia	4 (7.0%)	2 (6.7%)	4 (7.0%)	2 (6.7%)	0	0
Abdominal pain	12 (21.0%)	9 (30.0%)	12 (21.0%)	9 (30.0%)	0	0
abdominal distension	6 (10.5%)	4 (13.3%)	6 (10.5%)	4 (13.3%)	0	0
Ascites	2 (3.5%)	1 (3.3%)	2 (3.5%)	1 (3.3%)	0	0
Fatigue	3 (5.3%)	2 (6.7%)	3 (5.3%)	2 (6.7%)	0	0
Diarrhea	1 (1.8%)	0	1 (1.8%)	0	0	0
Edema	1 (1.8%)	1 (3.3%)	1 (1.8%)	1 (3.3%)	0	0
Increased alanine aminotransferase	23 (40.4%)	14 (46.7%)	21 (36.8%)	14 (46.7%)	2 (3.5%)	0
Increased aspartate aminotransferase	24 (42.1%)	11 (36.7%)	24 (42.1%)	11 (36.7%)	0	0
Increased blood bilirubin	13 (22.8%)	5 (16.7%)	13 (22.8%)	5 (16.7%)	0	0
BUN	5 (8.8%)	2 (6.7%)	5 (8.8%)	2 (6.7%)	0	0
White Blood cell	18 (31.6%)	8 (26.7%)	17 (29.8%)	8 (26.7%)	1 (1.8%)	0
Anemia	13 (22.8%)	6 (20.0%)	12 (21.1%)	6 (20.0%)	1 (1.8%)	0
Proteinuria	57 (100%)	28 (93.3%)	57 (100%)	28 (93.3%)	0	0

## 4 Discussion

Advancements in the understanding of the molecular mechanisms of HCC, along with the continuous development of novel therapeutic strategies, have increased interest in the combined application of TACE, TKIs, and ICIs. This comprehensive treatment approach is an innovative strategy against HCC, but its underlying mechanisms and therapeutic efficacy require further exploration. We assessed the use of TACE to control uHCC tumors, combined with TKIs and ICIs to achieve the goal of conversion therapy and facilitate R0 resection. Drawing upon existing research and comparing various conversion therapy strategies, the TACE+TKI+ICI has demonstrated promising rates of successful conversion (36). TACE is currently the preferred local treatment for uHCC. Its primary mechanism involves embolizing the tumor-feeding arteries to achieve local ischemia and facilitate deep penetration of chemotherapy drugs, thereby achieving local tumor control. However, some patients may not achieve the desired outcomes when TACE is used as a single treatment (37). TKIs, particularly sorafenib and lenvatinib, act primarily by inhibiting the target tyrosine kinase pathways associated with tumor growth and angiogenesis, thereby halting tumor progression (38). ICIs, in particular PD-1 inhibitors, operate by restoring the suppressed T-cell function inhibited by tumors. However, there is variability in the response of patients to ICIs, probably due to differing tumor microenvironments (39). As an initial treatment, TACE directly damages tumor tissues, leading to the release of vast amounts of tumor antigens. This provides the necessary targets for ICIs to activate the immune system (40). Simultaneously, TKIs inhibit the reactive angiogenesis that may be triggered after TACE treatment, while also reducing the blood supply to the tumor (20). ICIs lift immune suppression, allowing T cells to effectively recognize and attack tumor cells. Moreover, the environment created by TACE and TKIs further enhances the immune response (41). As a result, the triplet therapy should synergistically facilitate effective conversion therapy for uHCC. Compared to single treatments, the triplet therapy with TACE+TKI+ICI seems to significantly improve conversion rates. Thus, a substantial proportion of patients undergoing this combined therapy should experience a reduction in tumor volume. Moreover, according to previous researches, the triplet therapy has demonstrated a clear advantage over monotherapy or doublet therapy in prolonging the OS and RFS of uHCC patients (42). According to the literature, conversion therapy of uHCC with triplet therapy leads to higher conversion rates in patients with BCLC stage A/B than in those with stage C; with stage A providing the best outcome, followed by stage B, and then stage C (43). However, in the clinical practice, the focus is primarily on stages B and C, and the conversion rate of patients with BCLC stage B is higher than that of patients with BCLC C. However, the triple conversion therapy did not demonstrate a significant improvement in RFS or OS compared to other treatment strategies. This may be attributed to the limited sample size and inconsistent follow-up durations. We aim to address these factors in future prospective randomized controlled trials. Meanwhile, some studies have found no significant differences in RFS among stages A, B, and C (44). Therefore, multicenter prospective studies are needed to explore the impact of BCLC staging on uHCC conversion therapy. Studies have shown increased risks of adverse events with the addition of treatment modalities; however, these are generally manageable. TACE+TKI+ICI therapy may induce some AEs, but most are mild and manageable. The most common AEs include hypertension, thyroid dysfunction, hepatic impairment, gastrointestinal reactions, and skin rashes (45). These reactions are mostly temporary and can be managed through medication adjustments or dose reductions. Compared with monotherapy, triplet therapy is more likely to result in adverse events such as neutropenia, hepatotoxicity, and embolic events (46). Therefore, the short-term management for patients involves frequent blood count monitoring and regular liver function tests during treatment, whereas the long-term management requires monitoring of cardiovascular risks and further lifestyle interventions. For patients initially deemed unsuitable for surgery, the triplet therapy facilitates tumor reduction and may also render some eligible for surgical resection. Therefore, the TACE+TKI+ICI strategy presents a novel and promising direction for HCC treatment. Of course, some studies have argument in triplet therapy. For example, Kudo’s study showed that although triplet therapy may theoretically provide strong efficacy; in actual clinical trials, the effects of dual therapy can be sufficient, and further increasing the treatment modalities (such as adding TACE or additional TKIs) may not necessarily bring additional benefits (46). Therefore, multicenter, prospective, randomized controlled trials are needed to investigate the therapeutic effects and AEs of triplet therapy versus monotherapy or dual therapy. Studies on the markers for predicting the therapeutic effect of triplet therapy are also needed. We plan to screen for effective efficacy markers in the research of triplet therapy in future studies.

This study has some limitations. First, the patients with uHCC diagnosed at Shanghai Eastern Hepatobiliary Surgery Hospital often present with relatively poor baseline conditions. Therefore, the achieved ORR and CR after combination therapy were not as satisfactory as anticipated. And the success rate of conversion is improved, but the OS and RFS were not particularly improved. This could also be due to the relatively small sample size in our study and differences in treatment modalities. Therefore, randomized controlled trials with large samples are needed to validate our findings. Additionally, our clinical study lacked direct comparisons between the various treatment modalities. Instead, we compared our data with previous relevant studies, the triplet therapy showed significant benefits. In our experience, the adverse effects of the TACE+TKI+ICI strategy were mostly manageable, and we believe these risks are acceptable given the benefits of the therapy. The short follow-up period is another limitation of our study. We plan to conduct multicenter randomized controlled trials with long-term follow-up to further evaluate the long-term prognosis and AEs of triplet therapy. Finally, the therapeutic drugs used in this study were not standardized enough. Triplet therapy can be administered with various medications determined according to the clinical experience of the physician prescribing it. Studies are needed to evaluate the efficacy and safety of different treatment methods.

## Data Availability

The raw data supporting the conclusions of this article will be made available by the authors, without undue reservation.

## Supplementary material

Supplementary Figure 1 Flowchart of the Literature studies selection process.

Supplementary Figure 2 Contrast-enhanced CT scans of HCC lesions before and after treatment.
