# Correction: Conversion therapy with TACE, TKIs, and ICIs for unresectable BCLC stage B and C hepatocellular carcinoma

**DOI:** 10.3389/fimmu.2025.1647890

**Published:** 2025-06-30

**Authors:** Zhuoran Wang, Cunzhen Zhang, Jianhua Yin, Nan Li

**Affiliations:** ^1^ Department of Hepatic Surgery I (Ward I) Shanghai Eastern Hepatobiliary Surgery Hospital, Navy Medical University, Shanghai, China; ^2^ Department of Epidemiology, Faculty of Navy Medicine, Navy Medical University, Shanghai, China; ^3^ Key Laboratory of Biological Defense, Ministry of Education, Shanghai, China; ^4^ Laboratory of Medical Bioprotection, Navy Medical University, Shanghai, China; ^5^ The Second Affiliated Hospital, Wenzhou Medical University, Wenzhou, Zhejiang, China

**Keywords:** hepatocellular carcinoma, TACE, anti-angiogenic therapy, anti-PD-1 antibody, conversion therapy

In the published article, there was an error in the author list, and author “Cunzhen Zhang” was erroneously omitted as equal contributing first author. The corrected author list appears below.

“Zhuoran Wang^1,2,3,4†^ Cunzhen Zhang^1†^ Jianhua Yin^2,3,4*^ Nan Li^1,5*^

^†^These authors have contributed equally to this work and share first authorship”

The original article has been updated.

