# Supplementary figures and images for: Conversion therapy with TACE, TKIs, and ICIs for unresectable BCLC stage B and C hepatocellular carcinoma
Source: Front Immunol. 2025 Jun 10;16:1451965. doi: 10.3389/fimmu.2025.1451965 (PMC12185523; doi:10.3389/fimmu.2025.1451965)

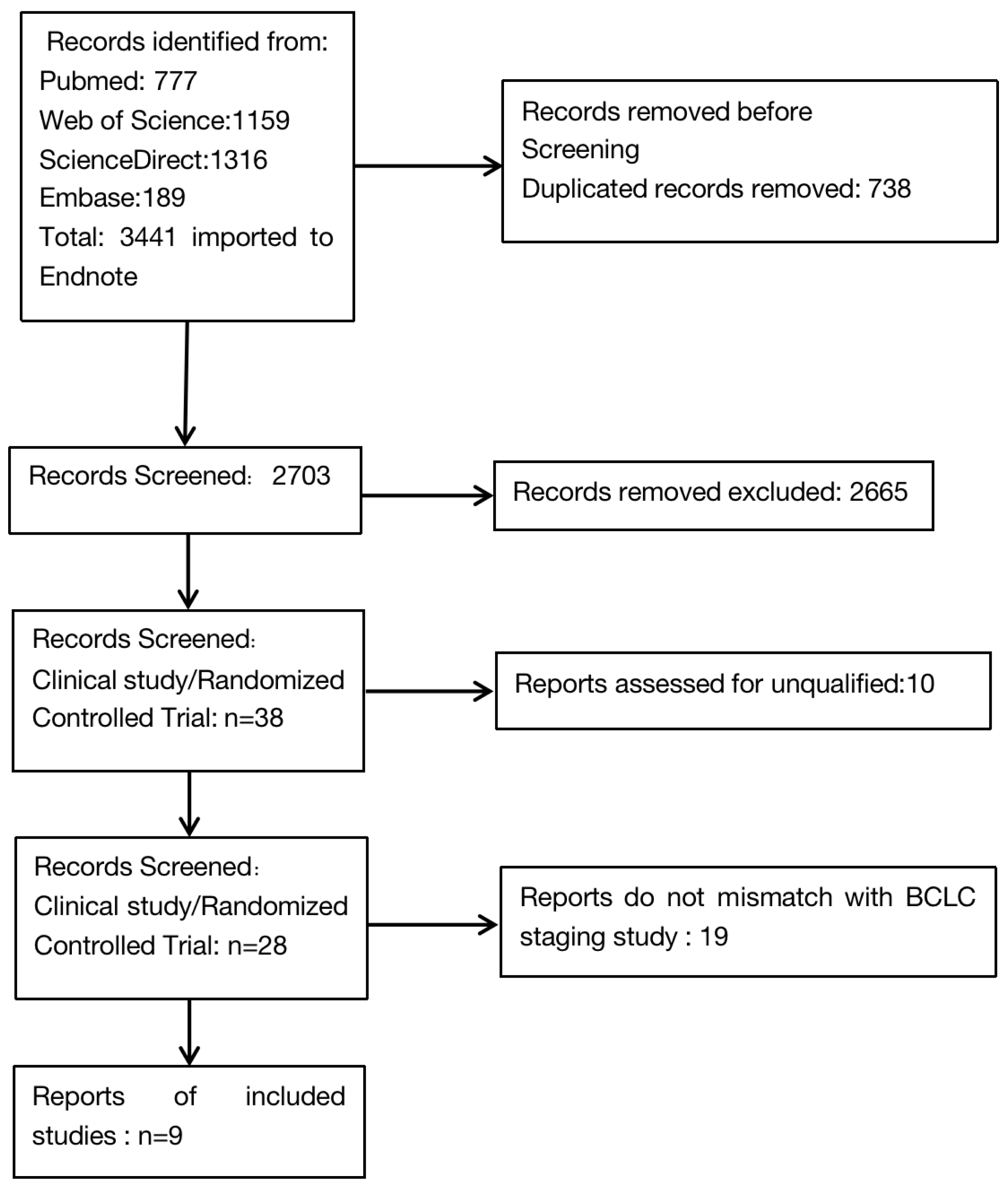

Supplement: Supplementary Figure 1 — Flowchart of the Literature studies selection process. [file Image1.tif]

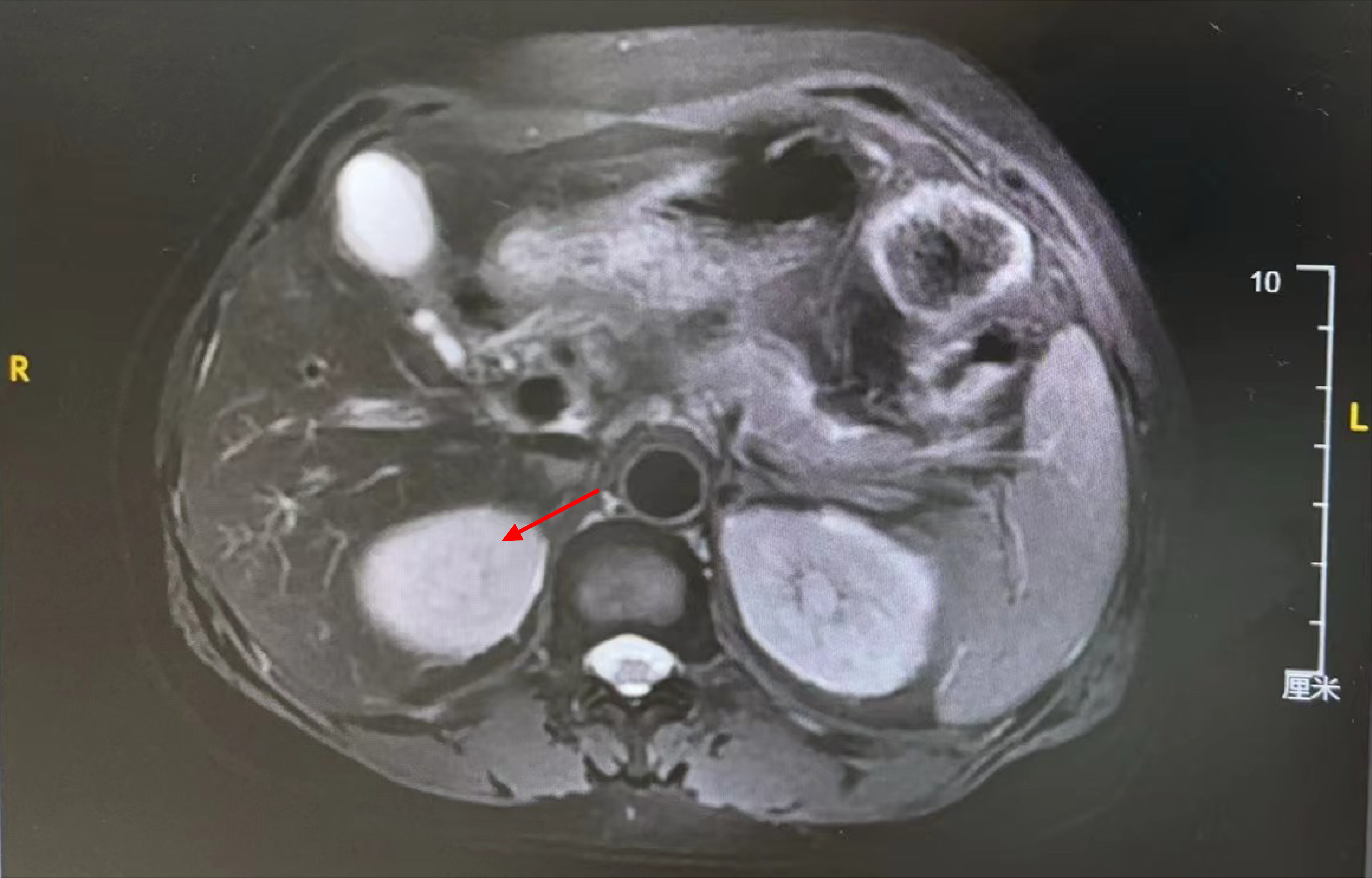

Supplement: Supplementary file 6 [file DataSheet5.zip › patient3 pre treatment.tif]

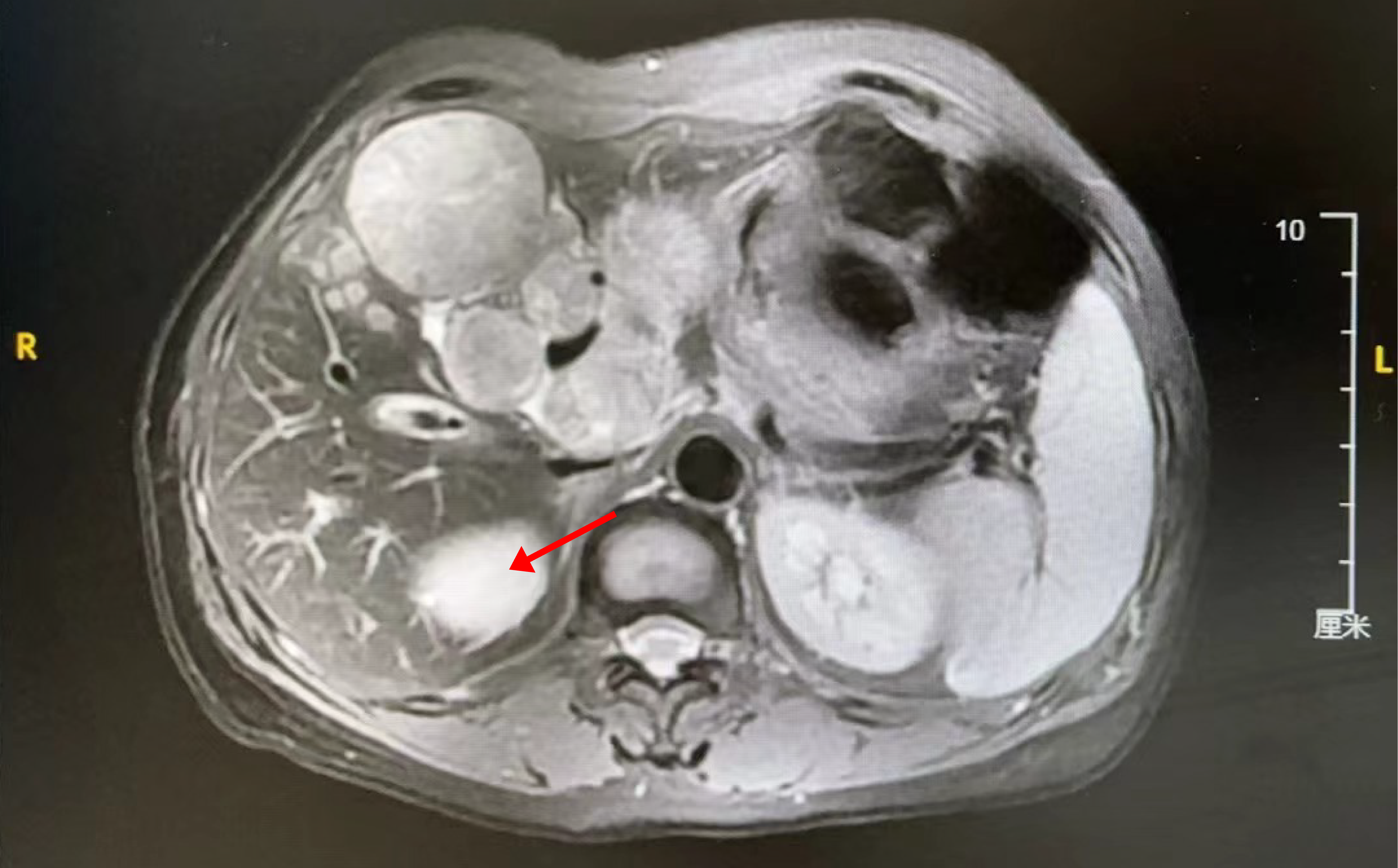

Supplement: Supplementary file 7 [file DataSheet6.zip › patient3 post treatment.tif]

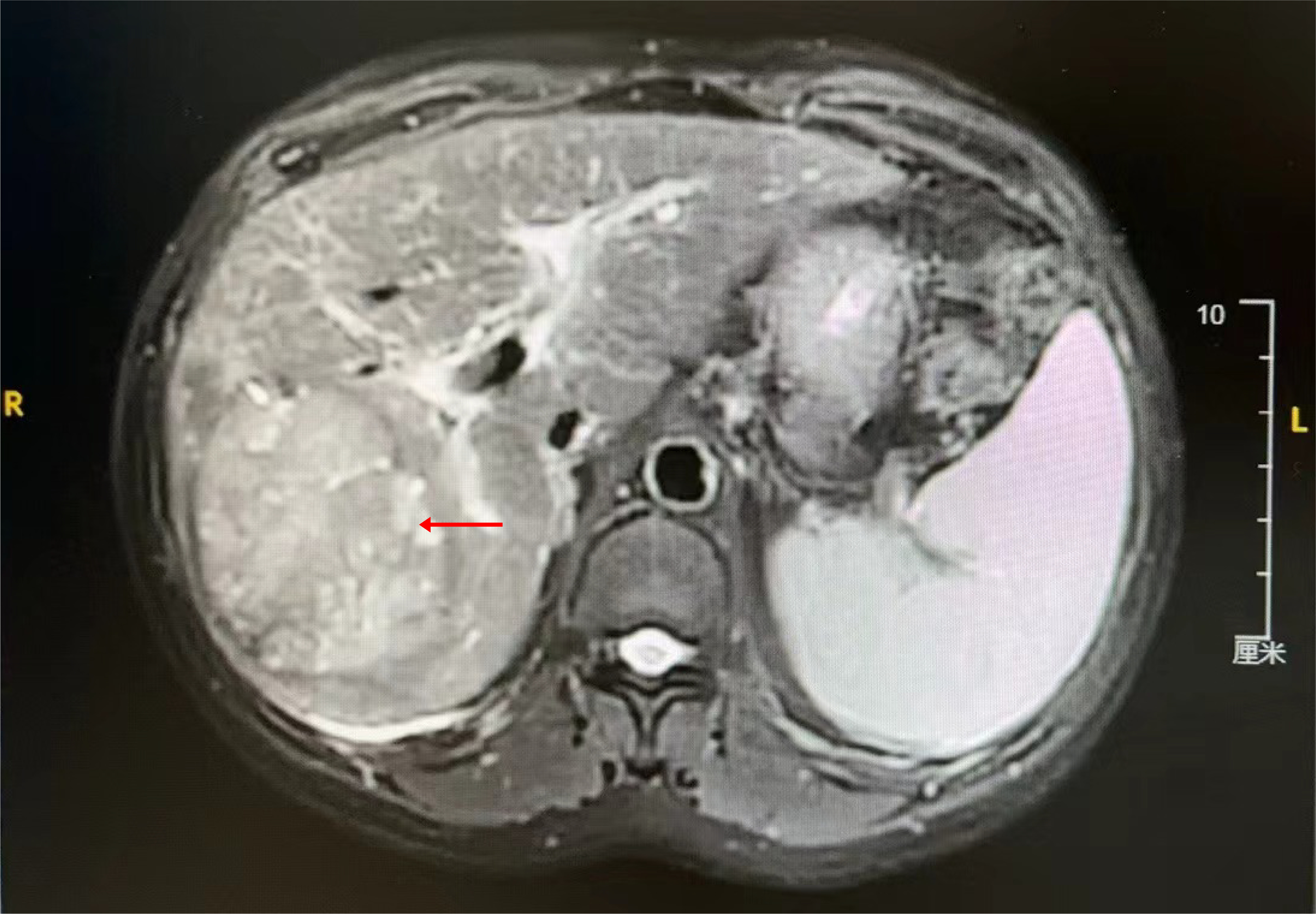

Supplement: Supplementary file 9 [file DataSheet8.zip › patient4 post treatment.tif]

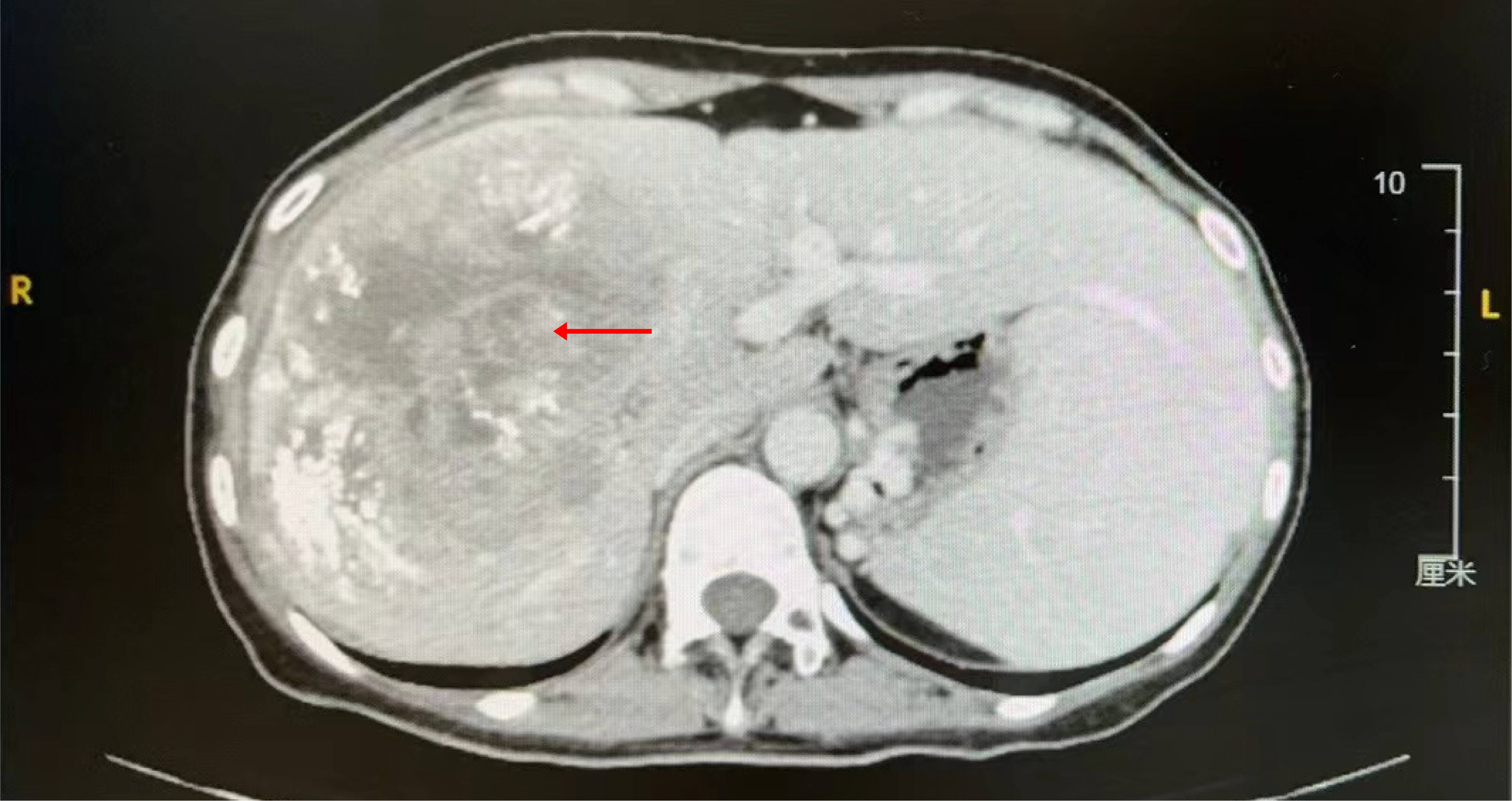

Supplement: Supplementary file 12 [file DataSheet11.zip › patient6 pre treatment.tif]

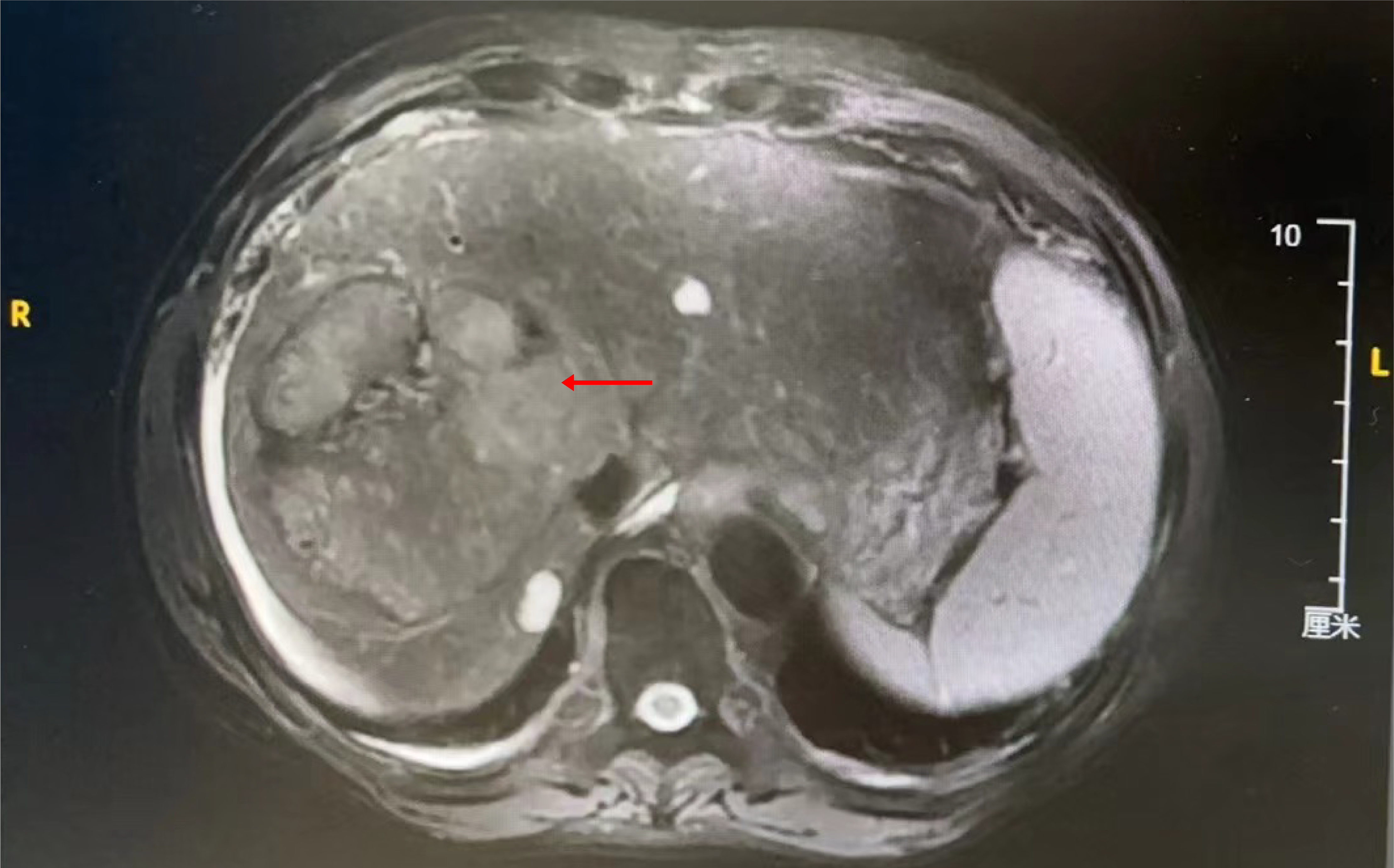

Supplement: Supplementary file 14 [file DataSheet13.zip › patient7 pre treatment.tif]

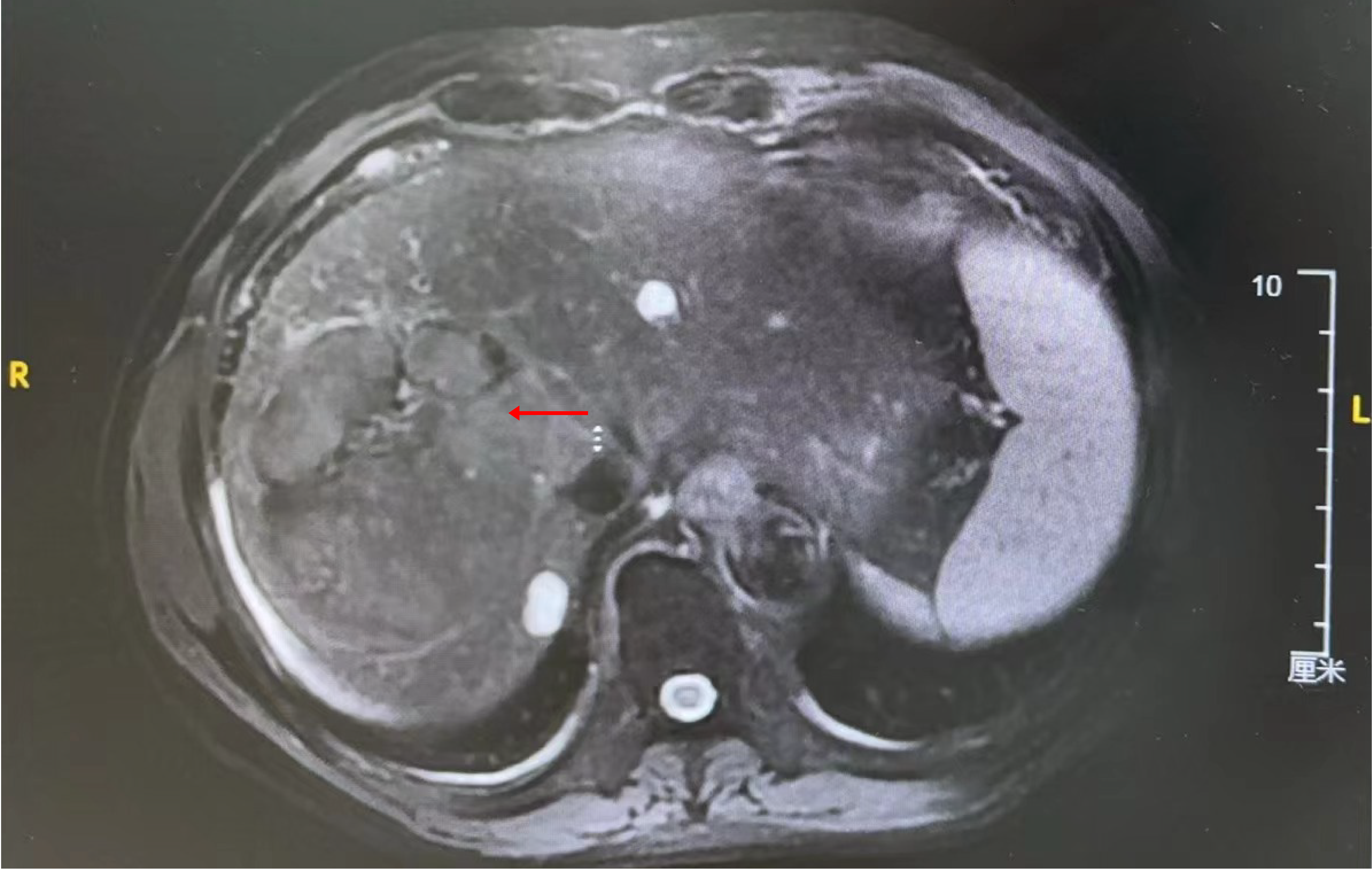

Supplement: Supplementary file 15 [file DataSheet14.zip › patient7 post treatment.tif]
